# Mr. Davies, on Hydro-Thorax

**Published:** 1800-11

**Authors:** H. Davies

**Affiliations:** Piccadilly


### *To the Editors of the Medical and Phyfical Journal.*

GENTLEMEN,

I Have read with confiderable pleafure, in a former Number of your extenfively ufeful Journal, a paper by Dr. Magennis, on his treatment of dropfical cafes*. His preliminary obfervations on the accuftomed plan of treating that difeafe appeared to me fo well founded, his arguments in favour of a different one fo conclufive, and the mode he has adopted fo well calculated to infure fuccefs, that I was fully refolved to try its efficacy in the firft cafe that occurred of the dropfical kind, and which happened in a very fhort time after I had read the account; the refult of which I beg leave to communicate.

I was called to attend an aged lady, about 70, who had laboured under an Hydro-thorax for fome months; fhe had a fmall but quick and very irregular pulfe, great palpitation on taking

* Vol. IV. page 212.

the leaft exercife, and was incapable of remaining in the horizontal pofture at night above two hours; when fhe was fuddenly feized by a fenfe of fuffocation, and was conftrained to rife and fit in her chair to pafs away the remaining dreary hours; her breath was exceedingly fhort, her appetite very much impaired, and her whole frame in an emaciated ftate. She had been under the ufual mode of treatment for fome time, but with very little fuccefs; indeed, from her advanced period in life, and the particular nature of that fpecies of dropfy, the cafe wore a very unpromifing afpect, as it fcarcely needs be obferved how feldom that difeafe yields to the moft rational treatment, and under the moft favourable appearances.

I began to exhibit the medicine much in the fame form and dofe as Dr. M. mentions in his own practice, only fubftituting the *ammon. pp.* for the *fixed alkali*, and the *mift. camph.* as the bafis inftead of the *aq. menth. fat.* which I thought more congenial to my patient's conftitution.

On calling the following day I was much pleafed to find, that after having taken a dofe of the medicine every fix hours, it had perfectly agreed with the ftomach, and fome little increafe had been obtained as to the quantity of urine voided; this I thought a favourable fymptom, and I was encouraged to perfevere in the ufe of the fame medicines with a very fmall augmentation of their dofes; and as I expected that the principal relief was to be obtained by a determination to the kidneys producing a copious flow of urine, I defired the nurfe to be very particular in afcertaining the quantity of water evacuated every day, and likewife that of liquids taken into the fyftem in the fame given time. I found upon enquiring into thefe two circumftances the following day, that about *four pints* of urine had been evacuated, and not above a *pint* of liquids drank; and had the happinefs of finding my patient materially relieved. She had been able the preceding night to lie in bed, and obtained fome reft; her breath was confiderably liberated, the palpitation very much decreafed, and the anafarcous fwellings of the legs (which I had omitted to mention in the firft account of her cafe) greatly diminifhed. Having every encouragement to proceed, I perfifted in the ufe of the remedies without any alteration, excepting a very inconfiderable addition to the quantities of the active ingredients, and found for the two or three following days nearly the fame evacuation as to urine, bearing a fimilar proportion to the liquids taken as mentioned above. It appeared to me now, from the abfence of the fymptoms attendant on Hydro-thorax, that the difeafe was fubdued, and the water collected in the cheft drained away; for the breathing was uninterrupted, and the palpitation entirely ceafed; therefore therefore I thought it unneceſſary to perſiſt in the *diuretic plan* any longer. But notwithſtanding the diſeaſe itſelf was removed, I knew there was conſiderable debility left; and to obviate which, to fortify the ſyſtem by a courſe of powerful tonics, appeared to me the moſt effectual means of ſecuring the patient againſt a relapſe; conſequently, I ordered her the *decoct. cinchonæ* joined with *zinc. vitr.* &c. three or four times a day with the happieſt effects; for, in two or three weeks afterwards her appetite was quite reſtored, her general ſtrength recovered; and at this preſent time ſhe enjoys a ſtate of general good health, to which for many months before ſhe was an utter ſtranger.

Thus have I briefly ſtated a ſucceſsful caſe, the communication of which I could not with propriety withhold from public peruſal, hoping it may conduce to general utility in the treatment of a diſeaſe which has often baffled the ſkill of the moſt ſagacious in the profeſſion; and ſhould the inſertion of it tend to this ſalutary purpoſe, the writer will be highly gratified in having an opportunity of caſting in his mite to the treaſury of the public good. I am,

GENTLEMEN,

Your's, reſpectfully,

H. DAVIES.

*Piccadilly,*
*October* 14, 1800.

